# Online eye tracking and real-time sentence processing: On opportunities and efficacy for capturing psycholinguistic effects of different magnitudes and diversity

**DOI:** 10.3758/s13428-023-02176-4

**Published:** 2023-08-01

**Authors:** Yanina Prystauka, Gerry T. M. Altmann, Jason Rothman

**Affiliations:** 1 Department of Language and Culture, UiT The Arctic University of Norway, Tromsø, Norway; 2 Department of Psychological Sciences, University of Connecticut, Storrs, CT USA; 3 Centro de Investigación Nebrija en Cognición (CINC), University Nebrija, Madrid, Spain

**Keywords:** Webcam-based eye tracking, Tracker-based eye tracking, Sentence processing, Online research

## Abstract

Online research methods have the potential to facilitate equitable accessibility to otherwise-expensive research resources, as well as to more diverse populations and language combinations than currently populate our studies. In psycholinguistics specifically, webcam-based eye tracking is emerging as a powerful online tool capable of capturing sentence processing effects in real time. The present paper asks whether webcam-based eye tracking provides the necessary granularity to replicate effects—crucially both large and small—that tracker-based eye tracking has shown. Using the Gorilla Experiment Builder platform, this study set out to replicate two psycholinguistic effects: a robust one, the verb semantic constraint effect, first reported in Altmann and Kamide,  *Cognition 73*(3), 247–264 (1999), and a smaller one, the lexical interference effect, first examined by Kukona et al. *Journal of Experimental Psychology: Learning, Memory, and Cognition, 40*(2), 326 (2014). Webcam-based eye tracking was able to replicate both effects, thus showing that its functionality is not limited to large effects. Moreover, the paper also reports two approaches to computing statistical power and discusses the differences in their outputs. Beyond discussing several important methodological, theoretical, and practical implications, we offer some further technical details and advice on how to implement webcam-based eye-tracking studies. We believe that the advent of webcam-based eye tracking, at least in respect of the visual world paradigm, will kickstart a new wave of more diverse studies with more diverse populations.

## Introduction

As is the case for (psychological) science in general, testing of existing psycholinguistic theories of real-time language processing is highly dependent on empirical work from a rather small subset of well-documented languages, overly skewed towards Western, Educated, Industrial, Rich and Democratic (WEIRD) contexts where resources for experimental laboratories are more widely available (Henrich et al., 2010; Luk, 2022; Nielsen et al., 2017; Rad et al., 2018; Rothman et al., 2022). And yet, even in such contexts, collecting data from participants with restricted accessibility to research facilities, from populations that are inherently geographically dispersed, or from subjects with lower socio-economic status can be complicated and limited by the logistical requirements of in-lab testing. Without a doubt, powerful online-based methods would increase the pools of potential participants for virtually any study. They would also facilitate access for language processing research where and for whom it currently does not exist, be it for languages and their speakers that are un(der)represented or for researchers whose contexts do not permit the type of equipment needed for expensive lab-based research. Of equal importance and relevance is the issue of statistical sensitivity: researchers could reallocate resources needed for lab-based testing to enroll more participants to increase statistical power—financial resources aside, it is feasible to run many more participants, and in a shorter time, when they are recruited and run online than when run in the lab.

Given this increased accessibility to participant pools, there has been a silver lining to the necessity, brought about by the COVID-19 pandemic, for many more researchers to have shifted to online methods. With the start of the pandemic, behavioral research involving lab-based experimental work confronted a (temporary) crisis: in-person participant testing was suspended for an indeterminant amount of time. As more and more researchers felt the pressure to adjust, online tools, already in existence but underused by many, emerged naturally as a logical gap-filling opportunity. Since many labs had to shift at the same time, the COVID backdrop did not allow for the typical, progressive growth curve in the collective uptake of newer/alternative technologies across the field. There was no time to sit back and wait as other labs refined existing online tools or developed bespoke ones. Now that a critical mass of data and new(er) tools have emerged in such a compressed time period, an integral part of the process is to test the comparability of online tools relative to their in-house lab-based counterparts. Doing so is rather important, not least as there is nontrivial skepticism regarding the efficiency of online testing methods in psycholinguistics, particularly for those that were not widely used prior to the past few years—even when the technology was available, e.g., eye tracking as opposed to reaction time/grammaticality judgment. As such, the present paper joins a rather small cohort of studies attempting to provide evidence on the efficacy and comparability of experiments conducted in the lab using dedicated hardware (referred to as tracker-based or lab-based eye tracking, using EyeLink, Tobii, or equivalent systems) and experiments run online using a webcam (referred to as webcam-based) in the visual world paradigm (VWP), specifically with “looking while listening” designs (Degen et al., 2021; Slim & Hartsuiker, 2022; Vos et al., 2022)^1^. The present study joins these others in offering some promising news: online eye tracking can replicate in-lab effects—even, as we shall show, quite small effects.

We contribute to this important discussion focusing on novel issues, such as comparing psycholinguistic phenomena that exhibit different effect sizes within the same experiment and exploring issues related to statistical power to better determine the utility boundaries of online eye tracking. Uniquely, our data provide evidence showing that even extremely small, fine-grained linguistic effects found in lab-based eye tracking can be replicated online. Demonstrating this has nontrivial consequences; to the extent that online eye tracking would be able to replicate work done in the lab one would have anticipated large, robust effects to be good candidates for online replication. However, this would not entail that small effects would also be capturable using webcam-based eye-tracking methods. By juxtaposing two effect types—a large and small one—within the same sizeable participant sample, it was possible to explore whether online eye tracking is indeed powerful enough to capture very subtle processing effects. This question must be addressed to understand the real, scalable potential of webcam-based eye tracking in psycholinguistics broadly. Moreover, while we had sufficient resources to recruit a large number of participants (*N* = 220) for our exploratory analyses (which contributed to the present study being the largest comparative eye-tracking study we are aware of in terms of tested participants), we realize that not every lab will have equivalent resources. Thus, the present paper also reports power analyses to assess the extent to which the effects we observed—one large and the other small—did in fact require such a large sample of participants. Such analyses can help researchers (including us) estimate appropriate participant numbers for similar, future studies utilizing webcam eye tracking. Given that power analysis for mixed-effects models is still a relatively unexplored area which requires further investigation (Kumle et al., 2021), the present study compares two approaches to the analysis of statistical power. Our study was conducted in Russian (a language underrepresented in psycholinguistics, with significant morphosyntactic differences as compared with English), using the increasingly popular software package Gorilla (Anwyl-Irvine et al., 2020).

In what follows, we briefly discuss tracker- versus webcam-based eye-tracking technology. Then, we review some previous non-language work testing webcam-based eye tracking, introduce the visual world paradigm (VWP), and review existing online psycholinguistic eye-tracking studies. Finally, we describe two original studies conducted with tracker-based (in-lab) eye tracking that we (conceptually) replicated online, before unpacking the broader significance of these findings.

### Tracker-based versus webcam-based eye tracking

Most recent psycholinguistic eye-tracking experiments have been conducted in a lab setting using specialized tracker-based eye-tracking hardware. These tend to apply signal processing algorithms to a camera feed. The EyeLink system, for example, tracks both the pupil and corneal reflection to determine the angle of the eye. Such systems afford fine-grained spatial accuracy (down to 0.15° visual angle) and temporal resolution (up to 2000 Hz, e.g., with the EyeLink 1000 Plus eye tracker, although the sampling rate in psycholinguistic studies rarely exceeds 1000 Hz). Dual-Purkinje eye trackers (e.g., Fourward Optical Technologies) track different reflections of infrared light from the eye: from the cornea and (in the Fourward case) the back surface of the lens (the first and fourth Purkinje reflections). These eye trackers are an order of magnitude more accurate (0.016°). They illuminate the eye with infrared light. In contrast, webcam-based eye tracking works based on light in the visible spectrum (which means that it is highly sensitive to lighting conditions); its spatial accuracy^2^ is an approximately 4.16° visual angle (as tested in Semmelmann & Weigelt, 2018), and its (current) limit on temporal resolution is 60 Hz (Yang & Krajbich, 2021). An object that occupies four degrees of visual angle at a distance of 60 cm is approximately four cm wide (a reasonably accurate rule of thumb is that at 60 cm viewing distance, degrees of visual angle translate into centimeters of lateral distance). The resolution of the webcam affects both the number of pixels with which to model the eye on the one hand (the higher the resolution, the more pixels and the more fine-grained the resulting definition of the eye will be) and frame rate and latency on the other (in general, the higher the resolution, the slower the frame rate and latency, with latency referring to the delay between when the webcam captures the video and when it may display that video on a screen) (Jensen, 2022).

The fact that webcam-based eye tracking uses light in the visible spectrum means that it is highly sensitive to lighting conditions and changes in the participant’s position (among others). This raises questions about the best techniques for calibration procedures. Calibration is the process of establishing a mapping provided by the eye tracker (webcam) and the known coordinates on the screen. A multitude of studies comparing calibration techniques exist for lab-based eye trackers (e.g., Blignaut, 2017; Nyström et al., 2013; Pfeuffer et al., 2013), and there is already a small emerging literature strand comparing calibration practices for webcam eye tracking (Saxena et al., 2022, as well as the studies summarized below). Calibration matters because poor calibration results in lower fidelity data (that is, it becomes less likely that where the eye tracker reports the eye is looking is where it actually *is* looking), and hence greater noise in the data, and reduced generalizability of the results as well as lower chances of finding the effect(s) of interest. This in turn lessens the likelihood of replicability of the target behavior. While the study reported here does not directly compare different approaches to calibration, it nevertheless shows that even small effects observed in-lab do replicate given currently available calibration procedures and notwithstanding the inevitable uncertainties of the mapping between webcam eye trackers and the eyes they track.

The development of the JavaScript-based WebGazer.js eye-tracking library (Papoutsaki et al., 2016) and its ease of integration into any website make webcam eye tracking a promising new tool for psycholinguistic research. WebGazer.js includes an eye-tracking model which self-calibrates by tracking visitors’ interaction with the webpage and trains a mapping between the features of the eye and screen positions. It runs locally in the client’s browser, so no video data are transmitted to a server (WebGazer.js, n.d.). Moreover, the implementation of WebGazer.js in the Gorilla Experiment Builder–graphical user interface (GUI)-based experiment builder software (Anwyl-Irvine et al., 2020) permits the running of webcam-based eye-tracking studies using a fully GUI-based approach with no coding experience necessary^3^. Relatively speaking, such an easy user experience is likely to lead to a significant upsurge in webcam-based eye tracking.

### Previous work testing WebGazer.Js

Semmelmann and Weigelt (2018) were one of the first studies to validate the use of WebGazer.js for cognitive psychology research. They tested it in a simple fixation task (participants were asked to fixate on a dot), in a pursuit task (participants were asked to follow the moving target stimuli) and a free-viewing face perception task. This approach allowed them to estimate saccades up to the target, detect the pursuit of the target by the participants, and replicate the finding that Western observers fixate more on the eyes than on other parts of the face in a free viewing task. They noted, however, that the spatial accuracy and sampling rate of consumer-grade webcams was lower than that of specialized hardware. Thus, they did not recommend using webcam-based eye tracking for studies requiring very detailed spatial resolution (for example, reading studies) or very fine-grained temporal information, or for a small number of trials.

In another more recent study, Yang and Krajbich (2021) further tested the feasibility of WebGazer.js, this time for decision-making research. They adapted the WebGazer source code by removing some unnecessary computations which, according to them, consumed computational resources and degraded temporal resolution without providing much added value^4^. Yang and Krajbich were able to replicate previous in-lab findings demonstrating a relationship between gaze and choice in a decision-making task, while being able to maintain the sampling rate of 50 Hz.

Further work suggests that webcam-based eye tracking in combination with WebGazer.js is a promising new tool in areas such as online video education (Madsen et al., 2021), mental state assessment (Paletta et al., 2020; Greenaway et al., 2021), medical image interpretation training (Quen et al., 2021), and episodic memory (Calabrich et al., 2021). All studies point out that the gaze position data collected with webcams (as opposed to specialized hardware) is noisier; however, they also point out that given how promising it is, this technology will be enhanced going forward and thus has huge potential.

### WebGazer.Js and the visual world paradigm

The visual world paradigm has been a productive paradigm in psycholinguistics which led to a plethora of important discoveries (see, e.g., Huettig et al., 2011, and Salverda & Tanenhaus, 2017 for review). In this paradigm, participants are presented with a visual display and an utterance. The display most often contains either a visual scene or individual objects arranged on a display, and typically the utterance mentions at least one of the displayed objects. Through careful experimental manipulation and research design, tracking participants’ eye movements allows the researchers to study the activation of different kinds of information (conceptual, semantic, syntactic, phonological, etc.) as language unfolds (Kamide et al., 2003a and b; Yee & Sedivy, 2006; Kaan, 2014; DeLong et al., 2005). One significant advantage of eye tracking over other behavioral techniques is thus its temporal resolution, which allows for testing hypotheses regarding the timing of activation of information as language unfolds—something that is impossible with end-of-the-sentence button presses which capture the end product of sentence comprehension. And while these two sides of the same coin ideally overlap, we also know from research that this is not always the case. There is a vast literature suggesting that language processing happens incrementally, i.e., comprehenders use incoming information to narrow down the set of referents satisfying the accumulating constraints (Altmann & Kamide, 1999; Kuperberg & Jaeger, 2016). This often leads to activation of information in a predictive manner, i.e., before the referring expression is even uttered, which can be studied with time-sensitive measures such as eye tracking. Participants in VWP studies can either be instructed to just sit and listen or to click with the mouse on an object mentioned in the sentence. The advantage of these approaches is that participants are not asked to provide any metalinguistic judgments (Huettig et al., 2011), which can affect processing by implicitly encouraging participants to develop task strategies. This method can thus be used with populations of speakers who are not able to provide such information (e.g., children). It can also be used to study the interplay of vision, language, memory, and attention within the same paradigm and thus affords testing comprehensive theories encompassing multiple cognitive processes recruited for the task of language processing (e.g., Huettig et al., 2011).

To the best of our knowledge, Degen et al. (2021) was the first published study which set out to replicate the VWP effect obtained in a lab and reported by Sun and Breheny (2020) using webcam-based eye tracking. Degen et al. (2021) examined whether the processing of scalar inferences is slower than the processing of numerals. This study had five regions of interest: four located in the corners of a visual display and one in the center of the screen. Degen et al. (2021) recruited 183 native English speakers through Amazon Mechanical Turk (MTurk). They replicated the effects reported in the original study, but with a delay of 700 ms. The authors discuss three potential reasons for the delayed effect. First, they say that the facial detection method and regression models that WebGazer.js uses for making predictions about gaze location may be computationally too demanding leading to lower sampling frequency and lag in the presentation of audio and images. Second, they speculate that prediction accuracy might have been compromised because their images were too close to each other (each region of interest [ROI] in their study consisted of an image of a person and a small number (2–3) of items next to them, and there were four such ROIs plus an object in the center of the screen). Third, they had a single calibration procedure preceding the task with relatively high tolerance for error. They speculated that either lowering that tolerance or increasing the number of calibration checks throughout the experiment could make the data more accurate.

While the data from this study do suggest that the temporal dimension of webcam-based eye tracking is compromised, a subsequent study by Vos et al. (2022) offers some promising findings. Vos et al. (2022) set out to replicate a study looking at grammatical aspect and event comprehension (Minor et al., 2022,5). In a 2-by 2-design, they contrasted minimal pairs of sentences containing verbs in past progressive and simple past and presented participants with images depicting the same event in different stages: either ongoing or completed. The task was to choose the picture which better matched the sentences. The experiment was conducted in English; 35 native English speakers took part in the original study and 124 participants were recruited online (through Prolific) for the replication study. Vos et al. (2022) programmed their experiment in jsPsych, which utilizes WebGazer for its eye-tracking functionality. There was an initial calibration, followed by additional calibrations every 12 trials. Both the original and the replication study found an almost at-ceiling preference for the ongoing event in the past progressive condition, and no preference for either picture in the simple past condition. A cluster-based permutation analysis revealed that the onset of the effect in the past progressive condition was 500 ms after the verb onset in the original study and 550 ms in the online replication study. This 50 ms difference in the timing of the effect is a significant improvement relative to the findings reported by Degen et al. (2021), which the authors attribute to the adjustments made to WebGazer by jsPsych^6^ (these adjustments addressed the timing issue reported in the publication of Yang and Krajbich (2021) and were later introduced into the version of WebGazer utilized by Gorilla software). It is worth noting is that this effect was detectable with a sampling rate of 20.73 Hz.

Perhaps most relevant to the present work is the study by Slim and Hartsuiker (2022). Using PCIbex (online experiment builder, Zehr & Schwarz, 2018), which utilizes WebGazer.js for eye tracking, they ran two experiments, one of which was a fixation task and the other was an online replication of a VWP study by Dijkgraaf et al. (2017) (which in turn was based on one of the VWP studies by Altmann & Kamide, 1999) looking at predictive processing. In the fixation task, participants were asked to fixate their gaze on a fixation cross which appeared in one of the 13 positions on the screen. The results of this experiment showed that it took WebGazer.js approximately 400–500 ms to detect that the participants’ gaze settled on the target location. The study also found that calibration scores predicted both temporal and spatial accuracy. The second study was a replication of just the monolingual portion of Dijkgraaf et al. (2017), which examined anticipatory processing in monolinguals and bilinguals (the monolingual portion is, therefore, a conceptual replication of Altmann and Kamide’s (1999) demonstration of anticipatory eye movements, albeit with objects arranged around the quadrants of a display rather than in scenes as in the original study). Subjects listened to sentences, half of which had constraining verbs, i.e. allowed participants to identify a referent already at the verb such as when observing a scene with four objects (a scarf, cheese, a comb, and a barrel) and hearing *Mary knits a … ,* participants did not need to hear the end of the sentence to predict that it would end in “scarf” because that was the only knittable object in the visual scene. The other half of sentences had non-constraining verbs, e.g., *Mary loses a …,* which could combine with any of the objects in the display and thus did not permit prediction. The finding that people look at the target object already at the verb more in the constraining than non-constraining condition is a well-established, robust effect, which has been replicated many times. Slim and Hartsuiker (2022) replicated the effect of the verb type whereby the proportion of fixations on the target image was higher following constraining than non-constraining verbs; however, this effect emerged 700 ms after the verb onset, which is 200 ms later than in lab-acquired data (this study was conducted with the version of WebGazer.js which did not solve the timing issue). The effect size of the online-acquired data was 60% of the effect observed in the lab-acquired data.

Given these findings, Slim and Hartsuiker wanted to test the minimal number of participants required to detect an effect that is half the size of the one observed in a lab. The sample size of the online replication study was 90 participants, while the original study tested 30 participants. An explorative simulation-based power calculation showed that the online experiment would reach 80% power with 70 to 75 participants. These findings thus have a number of important implications for psycholinguists running webcam-based eye-tracking studies. The good news is that the spatial resolution of the webcam eye tracker is sufficient to discriminate gazes across the four quadrants of the screen. However, this particular study does report a time lag in the temporal resolution relative to the in-lab eye tracking, which the authors hypothesize is caused by individual variation among participants, the contexts in which they participate, and the internal processing speed of the WebGazer.js algorithm. Looking ahead, this latter issue, as more recent studies, including the present one, are showing, is being overcome as the technology improves. The authors also encourage researchers to test the difference in effect size in webcam- and lab-based VWP studies to help further improve recommendations for sample size in online studies, a point which we will address in our empirical work presented below. Another important observation is that the authors needed to recruit 330 participants to obtain a sample size of 90 participants, because not everyone was able to pass the calibration stage. Thus, a procedure needs to be put in place to ensure that participants take part in the experiment only when they have access to good lighting conditions.

Our own study builds on Slim and Hartsuiker (2022) in that it also replicates the verb semantic constraint effect; however—and critically—it projects beyond it because we additionally look at a smaller effect of lexical interference originally reported in Kukona et al. (2014) within the same set of subjects, using different software (Gorilla), and conducting the experiment in a different language (Russian).

### Effects of interest

The studies reviewed above thus suggest that webcam-based eye tracking is, in principle, a viable psycholinguistic tool. However, given the current paucity of this method’s use coupled with the high stakes implicit in its wide-ranging potential uptake, additional validation is required. Most first attempts at replication with webcam-based eye tracking have, rightly and responsibly, examined very robust effects—after all, if large effects cannot be replicated, there would be little hope for more subtle, smaller ones—but current studies cannot really speak to the actual generalizability of using webcam-based eye tracking to test for much smaller effects. By combining a replication of a replication, i.e., examining the verb semantic constraint (Altmann & Kaminde, 1999) as in Slim and Hartsuiker (2022), while also looking to replicate a much smaller effect in the same participants—the lexical interference effect observed by Kukona et al. (2014)—our data have the potential to simultaneously bolster confidence in the method’s overall potential (i.e., beyond robust effects, if we replicate both) or help identify useful parameters for its employment (e.g., in the case we only replicate the more robust verb semantic constraint). As a result, we ask the following hitherto unanswered question: Is webcam-based eye tracking a feasible technique for studying much smaller linguistic effects?

Kukona et al. (2014) presented participants with sentences based on Altmann and Kamide (1999) with the addition of a color word, i.e., *The grandfather will smoke the black pipe*. The visual display contained images of a black pipe, a brown pipe, a black hat, and a brown hat. The critical comparison was that between the looks to the distractors: the black hat and the brown hat. The authors found that participants fixated on the black hat more than the brown hat at the onset of the final noun (“*pipe*”), which they interpreted as suggesting that comprehenders are susceptible to purely bottom-up effects due to the lexical item “*black*” (which engendered looks to *anything* black) despite the fact that the black hat did not meet the sentential constraint, i.e., it was not smokable object. The effect was rather small: descriptively it amounted to approximately a 5% difference in gaze proportions (as reported in Kukona et al., 2014). The semantic constraint effects observed in the Dijkgraaf et al. (2017) study amounted to a difference in target gaze proportions that was closer to 20% (Dijkgraaf et al., 2017, Fig. 2). Our goal was to explore whether both the large semantic constraint effect and the smaller lexical effect could be detected by webcam eye tracking. Studying this effect in Russian (a prelude to a future study that, similar to Dijkgraaf et al., would explore the role of second language proficiency) adds the further challenge that the equivalent sentences in Russian do not have determiners^7^, rendering the delay between verb onset and target word onset shorter than in the corresponding English sentences.

## Method

### Participants

Two hundred fifty-three subjects consented to participate in the study on Gorilla and went through the calibration procedure (207 people were recruited from the crowdsourcing platform Toloka Yandex, while the rest were recruited through Prolific or a direct link). Of these, 26 subjects did not pass the calibration stage^8^ and seven participants withdrew from the study without finishing it. The data from 18 more people were excluded due to the low sampling rate (<5 Hz, see “Data preprocessing” section for more details). The data from 202 participants entered the final analysis. Thus, 20% of participants failed to pass through to the analysis phase (cf. the 73% who failed in Slim & Harsuiker, 2022). Participants were dominant native speakers of Russian and were paid $10 for their participation.

### Stimuli materials

**Fig. 1:**
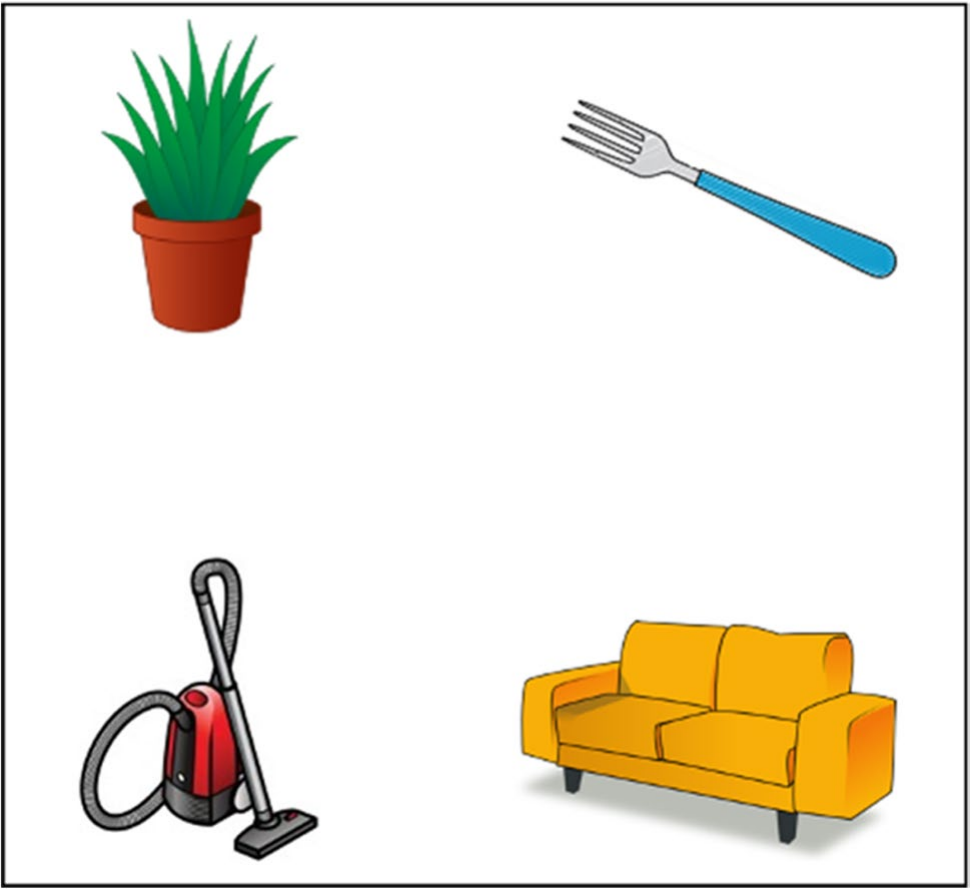
Example scene used to test the effect of verb semantic constraint. *Note.* Participants heard *Женщина польёт/подвинет растение* (The *woman will water/move the plant*)

The experiment included two sets of materials (intermixed) tested among the same participants within the same study. Materials for the replication of the semantic constraint effect included 16 sets of stimuli, each consisting of a quadrant-based visual scene with four images (see Fig. 1) and two minimal sentence pairs which differed only in the verb. Each sentence within a sentence pair belonged to one of the two conditions: constraining and non-constraining, based on the meaning of the verb with respect to the visual scene. Verbs in the constraining condition permitted only one of the four objects in the visual scene to be referred to post-verbally, e.g., *Женщина польёт растение (The woman will water the plant*). In the non-constraining condition, all four objects could be referred to post-verbally, e.g., *Женщина подвинет растение/вилку/пылесос/диван* (*The woman will move the plant/fork/vacuum cleaner/couch*); however, the target object was always the same as in the constraining condition. We constructed two lists of stimuli, each containing eight sentences in each condition (which is the same number of trials as in the original study), such that participants saw only one sentence from a minimal pair.

**Fig. 2:**
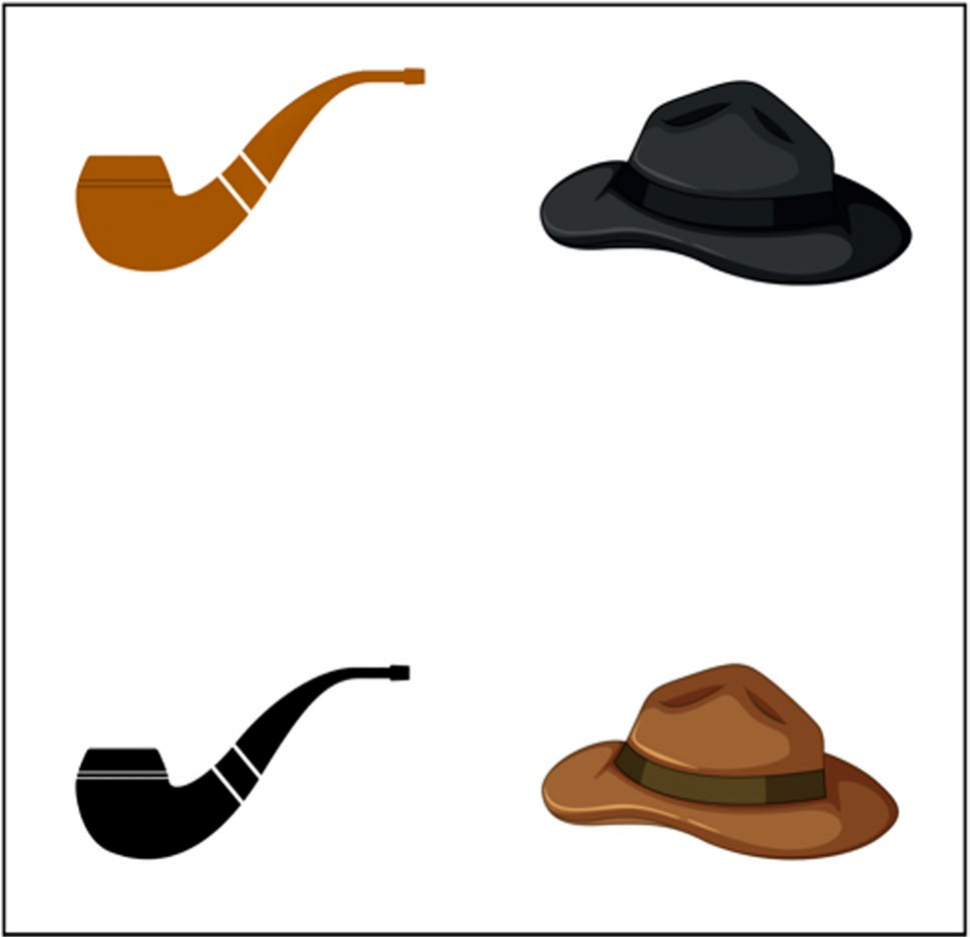
Example scene used to test the effect of lexical interference. *Note.* Participants heard *Дедушка выкурит эту черную трубку* (*The grandfather will smoke this black pipe*) or *Дедушка выкурит эту коричневую трубку* (*The grandfather will smoke this brown pipe*)

Materials for the replication of the lexical interference effect included 32 sets of stimuli^9^, each consisting of a quadrant-based visual scene with four images (see Fig. 2) and two minimal sentence pairs which differed only in the color adjective. Visual scenes consisted of two pairs of different object types, and objects from the same pair differed only by color, e.g., one visual scene could contain a black and a brown pipe and a black and a brown hat. All sentences had constraining verbs, i.e., only two of the four objects in a scene could appear in a postverbal position, and the target object could already be identified upon hearing the adjective. Each of the four objects in a scene thus represented one of the four conditions from a 2-by-2 design with the factors *verb consistency* and *color consistency*. For example, in our *pipe & hat* scenario, participants would hear a sentence *Дедушка выкурит эту черную трубку (The grandfather will smoke this black pipe)*. The black pipe is consistent with both the selectional restrictions of the verb and the color adjective, the brown pipe is consistent only with the verb, and not with the adjective, the black hat is consistent with the adjective, but not with the verb, and the brown hat is not consistent with either the adjective or the verb. To avoid any potential issue associated with the saliency of a particular color, half of the participants heard sentences with one color as a target, and the other half heard sentences with the alternative color as a target. Our analysis was focused on comparing the looks to the color-consistent and color-inconsistent distractors of a different type, i.e., the black and the brown hats in the example above.

There was also an additional factor of grammatical gender in this subset of stimuli, such that the target and the distractor either matched or mismatched in grammatical gender. However, since the epistemological point in focus for the present paper concerns the (potential) replication (or not) of the main effect of distractor type as reported in Kukona et al. (2014), we will not report the data from the analysis including the gender condition here.

A female native speaker of Russian (a professional voice actress) was instructed to produce the sentences naturally. Stimuli were recorded in a sound-attenuated booth and edited using Audacity software. Visual stimuli were created with the images obtained from the ClipArt collection (ClipArt, n.d.).

All materials are provided in Appendix A.

### Procedure

The experiment was programmed in the Gorilla Experiment Builder platform (Anwyl-Irvine et al., 2020) using the Eye Tracking Zone 2, which utilizes the updated version of WebGazer v2 accounting for the timing issue described above. Personal computers were set as the only allowed device type—i.e., no mobile phones—in both Gorilla and on the recruitment platforms (Toloka and Prolific). The study started with a video instruction explaining the purpose and the general procedure of the experiment, following which the participants were directed to the consent form. Participants who provided their consent were then redirected to the eye-tracking task, which started with more specific video instructions, example trials, and the first webcam calibration procedure. During calibration, participants were first presented with five red calibration points, one at a time, and were asked to fixate their gaze on each of them. After that, a validation stage followed where participants were presented with five green validation points, and were once again asked to fixate their gaze on them. In the validation stage, the eye-tracking zone tests its predictions: if it finds that predictions for the calibration points are closer to a different calibration point than to the target, the calibration fails and will be retried. Gorilla allows its users to choose the number of calibration point failures, which we set to 1 (so the strictest setting since it means that if validation fails for one out of five points, the entire calibration is considered failed and another round of calibration begins). We granted our participants three calibration attempts. In case of a third consecutive failed calibration attempt, participants were excluded from the experiment. Participants who successfully finished calibration started the eye-tracking experiment, which consisted of 48 trials (16 for the semantic constraint effect, 32 for the lexical interference effect, intermixed), split into three blocks, divided by two additional calibration routines (i.e., a new calibration occurred after every 16 trials). Each trial started with a fixation cross and proceeded to a visual display once the participants clicked on it. There was a preview time of 1000 ms, after which the audio was played (the actual audio onset time varied somewhat among participants and we return to this in the “Discussion” section). Participants were instructed to press on the object mentioned in the sentence after the sentence offset. The button press was activated after the audio offset. After the eye-tracking task, participants also performed a vocabulary task, a flanker task, and a grammatical gender task, as well as filled out a language background questionnaire. Given the present focus, however, results from these additional tasks are not reported here.

### Data preprocessing

The data files provided by Gorilla contain raw *x* and *y* pixel coordinates, as well as the coordinates in the normalized space. As discussed in the Gorilla documentation, the Gorilla layout engine lays everything out in a 4:3 frame and makes that frame as big as possible. The normalized coordinates are then expressed relative to this frame; for example, the coordinate 0.5, 0.5 will always be the center of the screen, regardless of the size of the participant’s screen. We used the normalized coordinates in our analysis. Gorilla provides two output data quality metrics. One of them is the mean convergence value (“convergence”) for fitting a facial model. This represents the model’s confidence in finding a face (and accurately predicting eye movements). Values vary from 0 to 1, and numbers less than 0.5 suggest that the model has probably converged. Another metric is “face_conf,” which represents the support vector machine (SVM) classifier score for the face model fit. This score indicates how strongly the image under the model resembles a face. Values vary from 0 to 1, and here numbers greater than 0.5 are indicative of a good model fit. There were no samples with values different from 0 on the “convergence” scale in our sample. Samples with values lower than 0.5 on the “face_conf” metric (mean 0.84, sd 0.37)—which together constituted less than 0.1% of the data—were removed.

**Fig. 3:**
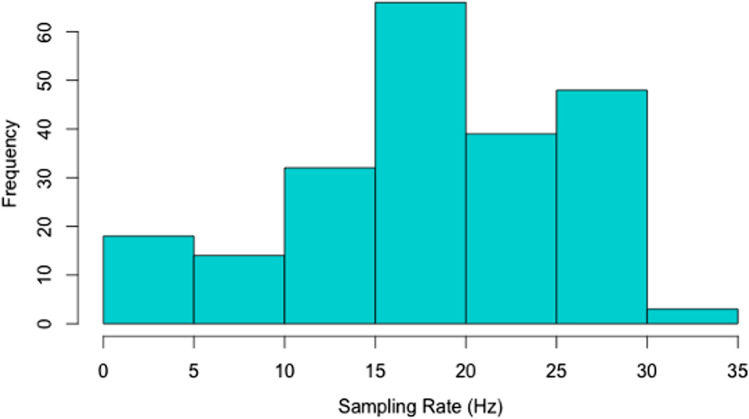
The distribution of participants’ sampling rate

The sampling rate of the original sample (*N* = 220) varied from 0.4 to 30.1 Hz (see Fig. 3 for the distribution of the sampling rate in our sample). We excluded participants with fewer than five samples per second, which resulted in the exclusion of 18 individuals. The mean sampling rate in the resulting group was 19.7 Hz (sd = 6.4 Hz, range 5.5 – 30.1 Hz).

**Fig. 4:**
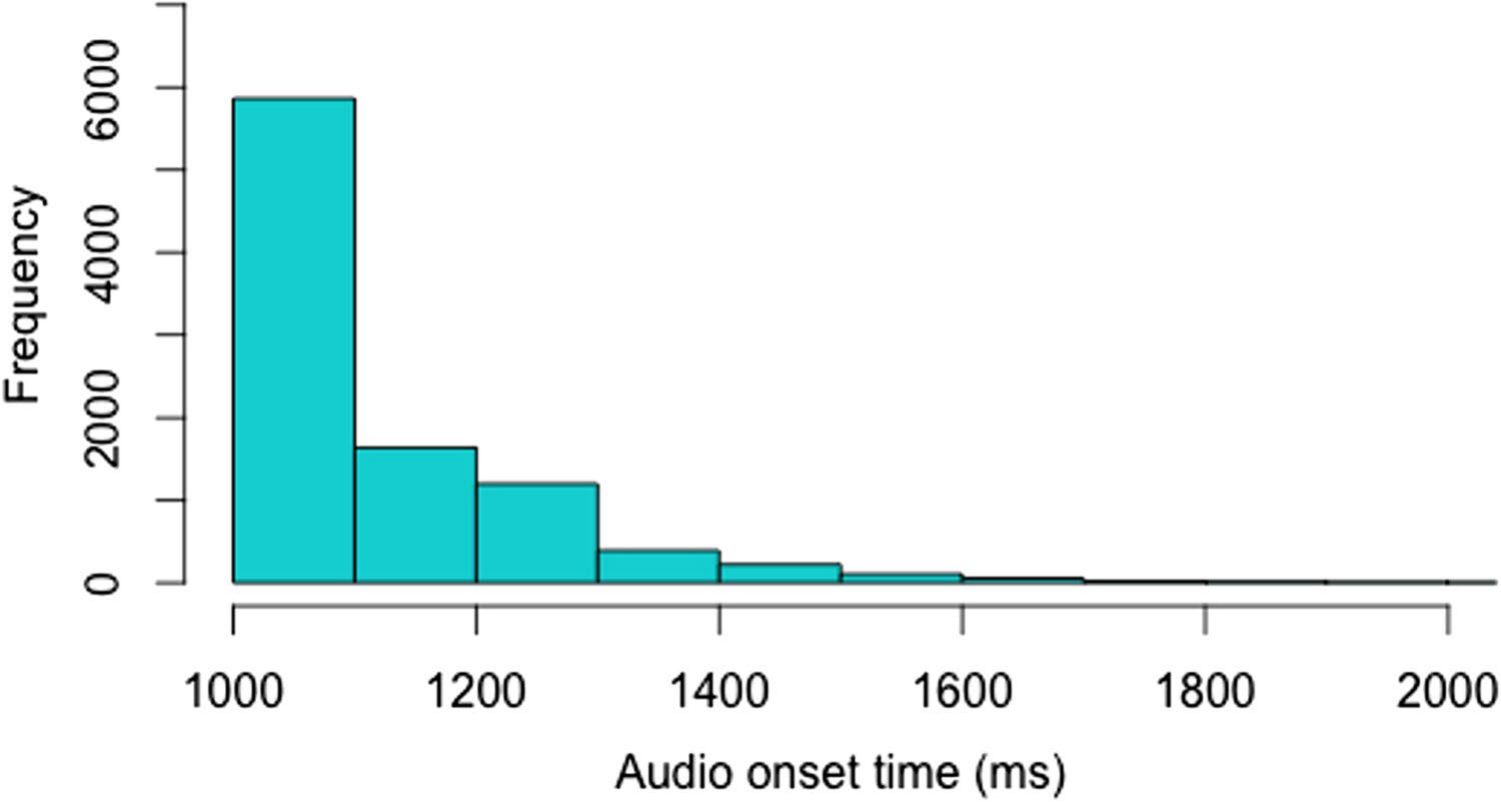
The distribution of audio onset times among participants

Additionally, the experiment design involved an image preview time of 1000 ms, although the *actual* onset time of the sentence varied between participants due to the properties of their hardware and connection speed. Gorilla provides an option for downloading additional metrics on audio events (a highly recommended option in the Audio Zone settings), and one of the metrics is the timing of when the audio event actually started (as opposed to when it was requested). The range of actual onset times was between 1001 ms and 23259 ms (there was a single value of a magnitude of this latter number, the second longest play time was 3487 ms, followed by the third with 2838 ms), with the mean of 1122 ms and the standard deviation of 245 ms. The density of onset times is plotted in Fig. 4. This information has a very important implication for researchers who are designing and analyzing their webcam-based eye-tracking study for computing the actual play times of critical words. So regardless of the software that one is using to run their experiments, access to a metric with actual onset times is essential. In our analysis, we accounted for such lags in the onset of the audio.

### Analysis

We expressed our regions of interest as quadrants. The one containing the target image (i.e., the object mentioned by the sentence) was the critical ROI in the Altmann and Kamide (1999) portion of the study, and the ones containing the distractor items (objects of a different type than the target, e.g., the brown and the black hat in the *“The grandfather will smoke the black pipe”* example) were the critical ones in the Kukona et al. (2014) portion of the study.

Linear mixed-effects modeling was used to analyze our data given that it is the current state of the art and can test for the effect of the condition while accounting for random effects of, e.g., trials or subjects. Following Kukona et al. (2014), the analysis focused on gaze locations at a single timepoint—the onset of the direct object noun. In the output provided by Gorilla, what is being measured is the first recorded gaze location following the onset of the target noun, which does not necessarily align with the noun onset. Given that different participants have different sampling rates, the timing between the actual noun onset and the first measured gaze location following it could differ among participants, however, crucially not too substantially (mean = 30.75 ms; sd = 22.24 ms, range: 0 ms to 99.9 ms 10). The average sampling rate of approximately 20 Hz in our sample means that participants’ looks were sampled every 50 ms, which is why we surmise that the standard deviation of 22 ms—deviations being an inevitable reality of online eye tracking—is within an acceptable window of variation that should not have a significant effect on the results in the relevant sense for our questions. The dependent variable was the number of trials with looks to the objects of interest at this timepoint.

#### Verb semantic constraint effect (Altmann & Kamide, 1999)

First, we computed the number of trials (per participant) with looks to the target at the onset of the direct object noun (e.g., “lemon” in “The woman will squeeze the lemon”). We then submitted these counts to the empirical logit transformation (as suggested by Barr, 2008), following this formula:

- *Empirical Logit <- log ((trials with looks to the target) / (total number of trials − trials with looks to the target + .5))*

Since we are using a linear approximation, we also computed the weights:

- *Weights <- 1/ (trials with looks to the target + .5) + 1 / (total number of trials − trials with looks to the target + .5))*

These transformed data were then submitted to the linear mixed-effects model with the fixed effect of Condition (verb type: constraining vs. non-constraining) and random intercepts for participants (random slopes were not utilized because there was only one data point per participant)^11^. The resulting model looked like this:

- *Model <- lmer (Empirical Logit ~ Verb Type + (1|Participant), Data = our data, weights=1/Weights)*

Finally, following Kukona et al., we used the model comparison approach to test for the significance of our fixed effect of verb type by comparing the model above to the base model:

- *Model <- lmer (Empirical Logit ~ (1|Participant), Data = our data, weights=1/Weights)*

#### Lexical interference effect (Kukona et al., 2014)

To test for the effect of lexical interference, we first computed the number of trials (per participant) with looks to the distractors of the same and different color at the onset of the direct object noun (the distractors were the objects of a different type than the target). We then submitted these counts to the empirical logit transformation:

- *Empirical Logit <- log ((trials with looks to the distractor + .5) / (total number of trials − trials with looks to the distractor + .5))*

The weights were computed in the following way:

- *Weights <- 1/ (trials with looks to the distractor + .5) + 1 / (total number of trials − trials with looks to the distractor + .5))*

These transformed data were then submitted to the linear mixed-effects model with the fixed effect of Condition (Distractor type: same or different color, relative to the adjective) and random intercepts for participants. The resulting model looked like this:

- *Model <- lmer (Empirical Logit ~ Distractor Type + (1|Participant), Data = our data, weights=1/Weights)*

We compared the model above to the base model without the fixed effect:

- *Model <- lmer (Empirical Logit ~ (1|Participant), Data = our data, weights=1/Weights)*

## Results

### Verb semantic constraint

**Fig. 5:**
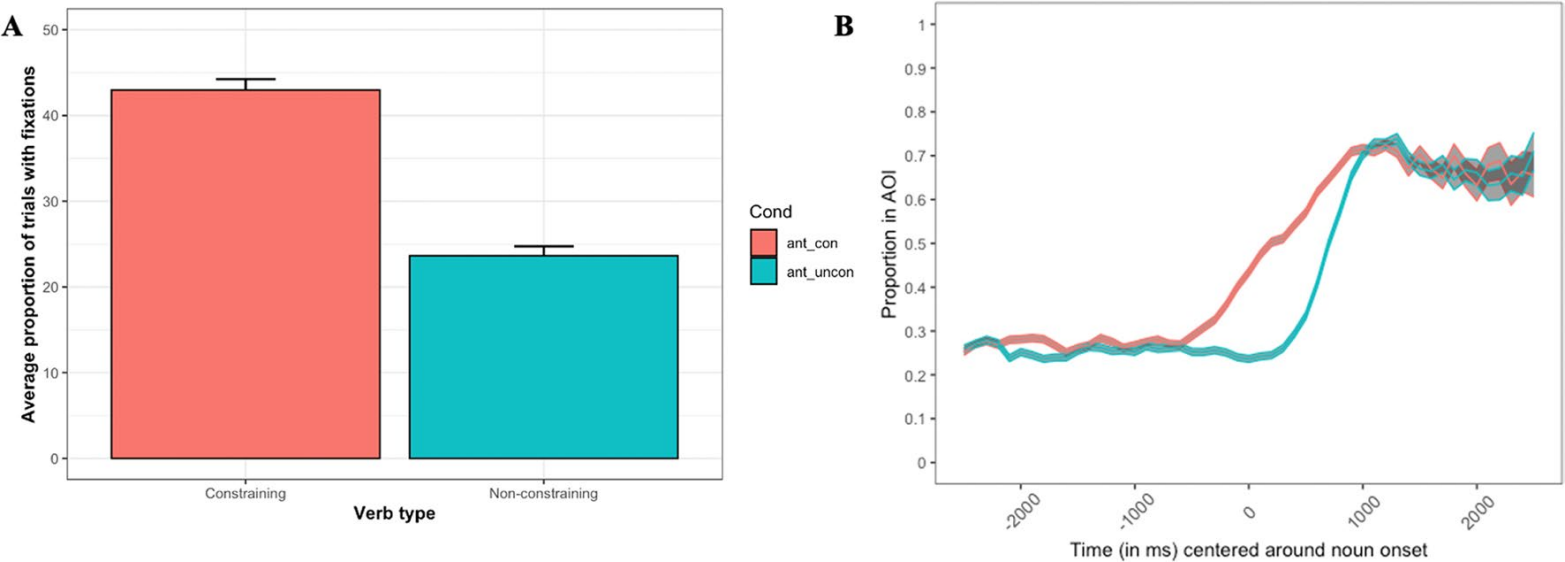
**A** The average proportion of trials with looks to the target following different verb types at the onset of a noun; **B** the average proportions of looks to the target across time. *Note.* A and B. Error bars and ribbons represent standard error

The average proportions of trials with looks to the target at the noun onset as well as the time course of the proportions of looks to the target are illustrated in Fig. 5. The results of the statistical analysis are summarized in Table 1.

**Table 1:** Summary of the model containing the effect of the condition (verb semantic constraint, columns 2-4) and of the base model without the effect of interest (columns 5-7).

*Predictors*	LME model with the effect of condition			LME base model		
	*Estimates*	*CI*	*p*	*Estimates*	*CI*	*p*
(Intercept)	-0.23	-0.31 − -0.14	**<0.001**	-0.52	-0.59 − -0.45	**<0.001**
Cond [DistractorType]	-0.65	-0.78 − -0.53	**<0.001**			
Random Effects						
*σ*^2^	0.73			0.91		
*τ*_00_	0.00_participant_id_			0.00_participant_id_		
N	202_participant_id_			202_participant_id_		
Observations	404			404		
Marginal *R*^2^/Conditional *R*^2^	0.128/NA			0.000/NA		

The analysis revealed a reliable effect of verb constraint (estimate = 0.65, SE = .06, χ^2^ = 91.02, *p* < 0.001, see Table 1), with more looks to the target following constraining verbs (average number of trials with looks to the target = 3.31, SE = 0.1, transformed = −0.28, SE = 0.05) as compared to non-constraining verbs (average number of trials with looks to the target = 1.82, SE = 0.08, transformed = −1.15, SE = 0.06).

**Fig. 6:**
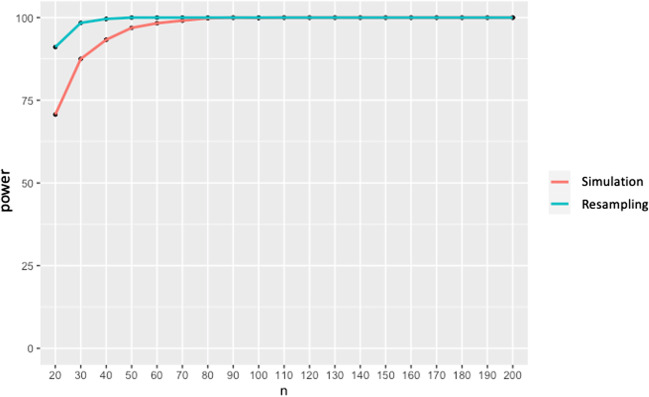
Results for power analysis for the Altmann and Kamide (1999) replication. *Note.* Power (*y*-axis) for the effect of verb type is plotted as a function of the number of participants (*x*-axis)

Findings from previous work (Slim & Hartsuiker, 2022) suggested that the effect size in a webcam-based eye-tracking study would be roughly half the size of in-lab experiments, thus necessitating more participants to gain sufficient power to detect the effect (Sullivan & Feinn, 2012). Our study is a conceptual, not a direct, replication of Altmann and Kamide (1999) given that it was conducted in a different language with different sentence and picture materials. Thus, the direct comparison of the effect sizes between the two is unwarranted. However, in order to provide estimates of the required sample size for similar experiments for future online studies, we ran some additional post hoc analyses to explore statistical power for different sample sizes. We used our relatively large sample size as an opportunity to compare two different approaches to power analysis: one simulation-based and one based on resampling. The simulation-based approach was performed using the *mixedpower* package in R (Kumle et al., 2021; the same approach was used for power analysis reported in Slim & Hartsuiker, 2022). Based on the data and the lmer model provided to *mixedpower,* it created new simulated datasets with the requested number of observations (we simulated new datasets for different sample sizes, from 20 to 200 in increments of 10^12^). These simulated datasets are based on the distribution expected by the model type, which in our case was Gaussian, and on the data structure captured by the model. *Mixedpower* then refitted the model and performed a significance test. It did so 1000 times per every sample size and calculated the proportion of significance of all simulations. The results provide an estimate of statistical power and are reported in Fig. 6. This power analysis suggests that we reach 80% power already with 30 participants to detect an effect of the observed size.

Our second resampling-based approach to power analysis was implemented based on the logic outlined by Strong and Alvarez (2019) and Rossman (2021) using custom R code (available on the Open Science Framework [OSF] page). The major difference from the *mixedpower* approach is that instead of simulating new datasets for the specified number of participants based on the distribution expected by *lmer*, we drew random samples of *N* participants (with replacement, which means that the same participant could be sampled more than once for the same sample) from our large dataset and ran a linear mixed-effects model for each sample (keeping the same model parameters that were used for the analysis). We did so 1000 times per sample size, with samples ranging from 20 to 200 in increments of 10. From each such analysis, we stored *t*-values for the main effect of Condition. We then counted the proportion of significant iterations (*t* ≥ 1.96) per each sample size. According to this analysis, one could reach 80% already with 20 participants (which means that out of 1000 iterations for random samples of 20 participants, >800 achieved significance).

### Lexical interference effect

**Fig. 7:**
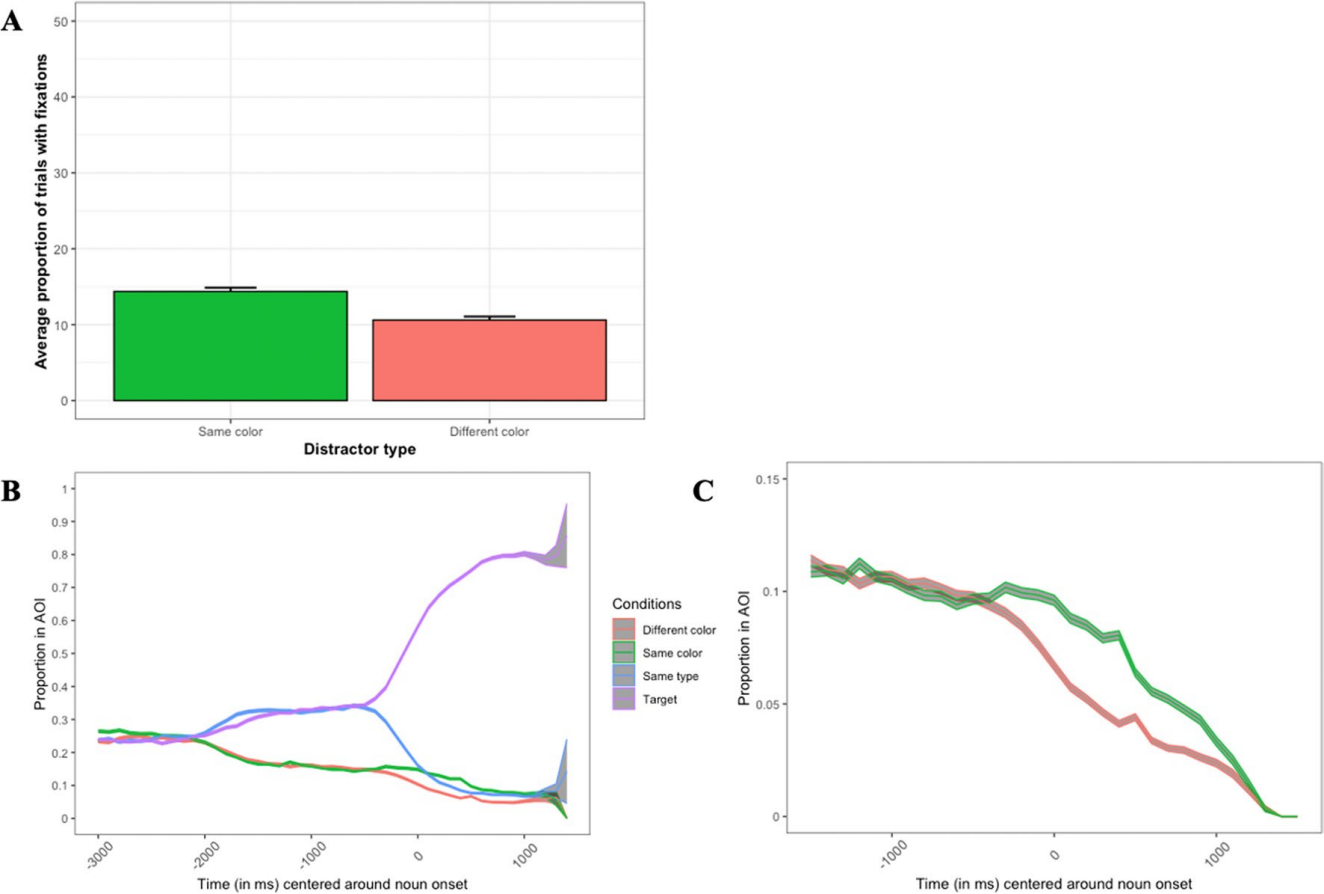
**A** The average proportion of trials with looks to distractors of different colors at the onset of a noun; the average proportions of looks to all objects in the display (**B**) and only to distractors in a zoomed-in window (**C**) across time. *Note.* A. Error bars represent standard error. B and C. Looks are time-locked to the onset of the noun. Error ribbons represent standard error

The average proportions of trials with looks to distractors as well as the time course of the proportions of looks to the distractors are illustrated in Fig. 7. The results of the statistical analysis are summarized in Table 2.

**Table 2:** Summary of the model containing the effect of the condition (distractor type, columns 2-4) and of the base model (columns 5-7)

*Predictors*	LME model with the effect of condition			LME base model		
	*Estimates*	*CI*	*p*	*Estimates*	*CI*	*p*
(Intercept)	-1.62	-1.69 − -1.55	**<0.001**	-1.72	-1.78 − -1.67	**<0.001**
Cond [ant_uncon]	-0.27	-0.36 − -0.18	**<0.001**			
Random Effects						
*σ*^2^	0.78			0.91		
*τ*_00_	0.05_participant_id_			0.03_participant_id_		
ICC	0.06			0.03		
N	202_participant_id_			202_participant_id_		
Observations	404			404		
Marginal *R*^2^/Conditional *R*^2^	0.022/0.080			0.000/0.029		

The analysis of distractors revealed an effect of adjective consistency (estimate = 0.27, SE = .05, χ^2^ = 29.64, *p* < 0.001, see Table 2), with more looks to adjective-consistent objects (e.g., the brown hat for the brown pipe example, average number of trials with looks to the brown hat = 4.30, SE = 0.15, transformed = −1.82, SE = 0.05) as compared with adjective-inconsistent objects (e.g., the black hat for the brown pipe example, average number of trials with looks to the black hat = 3.17, SE = 0.14, transformed = −2.19, SE = 0.05).

**Fig. 8:**
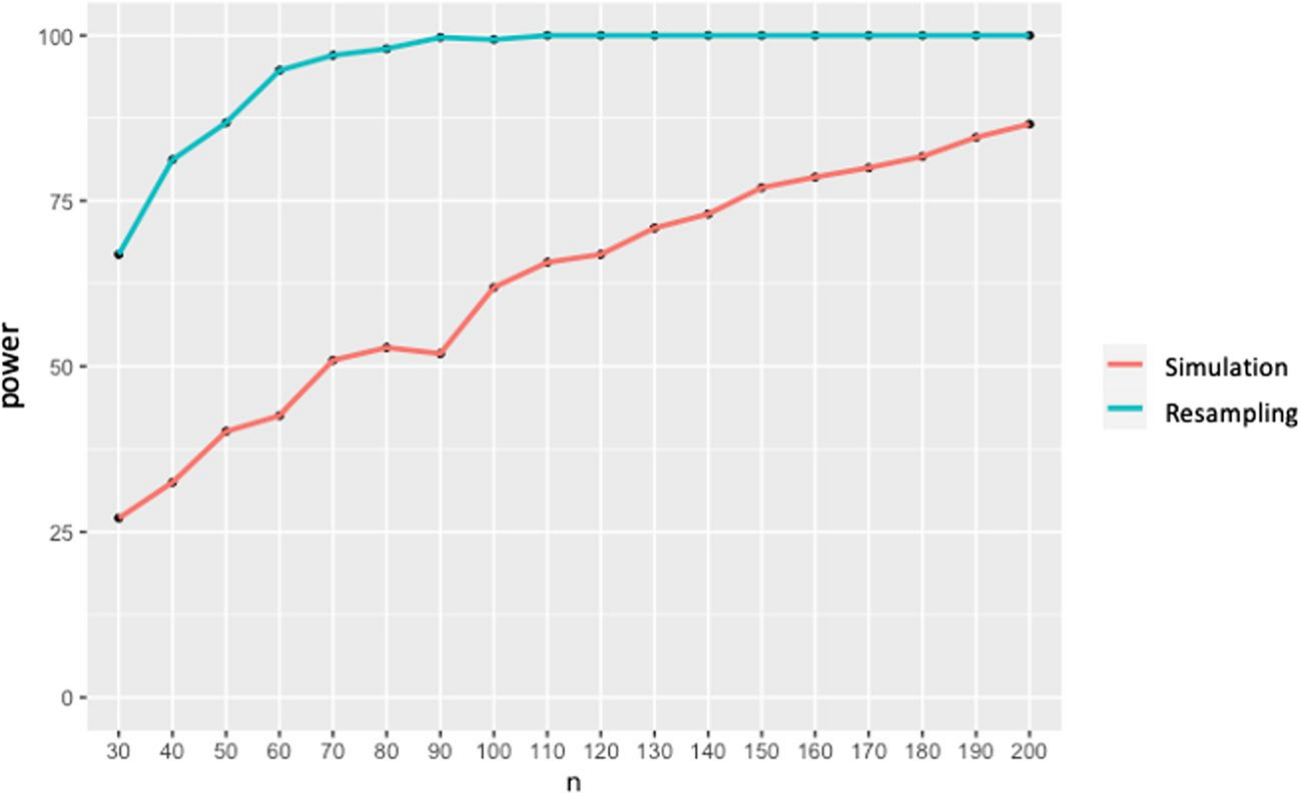
Results for power analysis for the Kukona et al. (2014) replication. *Note.* Power (*y*-axis) for the effect of distractor type is plotted as a function of the number of participants (*x*-axis)

Based on the model reported above, we simulated new datasets for different sample sizes (from 30 to 200 in increments of 10) and calculated the proportion of significance of all simulations. This power analysis suggests that we reach the 80% power with at least 170 and more participants to detect an effect of the observed size. The resampling-based approach resulted in a drastically different outcome, suggesting that a dataset with as few as 40 participants would reach 80% power. The results are reported in Fig. 8. We discuss this discrepancy between the two approaches in the Discussion section.

## Discussion

Our results add credence to the utility of webcam-based eye tracking as a viable alternative to its in-lab counterpart. We not only replicated the robust effect of the verb semantic constraint (Altmann & Kamide, 1999), replicating the replication of Slim and Hartsuiker (2022), but crucially also showed that webcam-based eye-tracking functionality is not limited to large effects as has previously been demonstrated. Notwithstanding the small size of the original effect, we also replicated the lexical interference effect of Kukona et al. (2014) within the same set of participants.

This latter replication is an important and novel contribution. Our paper shows, for the first time, that the domains for use of online eye tracking can be expanded in scope to more nuanced effects, and with some confidence. Crucial to our conclusion are our power analyses. We compared two approaches: one based on simulation (Kumle et al., 2021) and the other on resampling (Rossman, 2021; Strong & Alvarez, 2019). We found strikingly different results, especially for the small effect of lexical interference. Specifically, the *mixedpower* (simulation) approach suggested that we would reach 80% power with 170 participants, while the resampling-based approach showed that one would need as few as 40 participants (randomly selected from the original dataset). This discrepancy leaves us with the question of which approach to rely on, and under what circumstances. The major difference between the two approaches is how they handle the creation of new datasets. *Mixedpower* utilizes the lmer4::simulate() function, which uses the estimated model parameters to simulate new datasets by drawing random values from the corresponding distributions. These simulated data do not perfectly match the original data as they are generated from probability distributions rather than exact values of the original data. As such, they are dependent on the quality of the model. That is, they are contingent on the fit to the original data; the less variance accounted for by the model, the less representative of the actual distribution will be the simulated distribution on which power is calculated.

In the resampling approach, instead, we are drawing subsets of random subjects from the original dataset, thus testing the properties of the actual data distribution. By comparison, the *mixedpower* approach is not only more conservative, but also provides an arguably more generalizable estimate of power for populations which do not closely resemble the original dataset. Detailed discussion of which of these methods might be more useful for generalizing to future studies is beyond the scope of this paper. Nonetheless, there are useful observations to be made. First, power calculations are not simply useful prospectively—that is, for estimating required participant sample sizes in future studies. They are useful for understanding existing data. In the present case, they tell us that the effects we observed of verb semantic constraints are sufficiently robust across participants that we functionally replicated that aspect of the study several times over within the same study. In this sense, the resampling method is particularly suitable as we could essentially test different sized subsets of our participant pool, showing that in 80% of our samples sized *N* = 20, we would see the same effect. Thus, we effectively replicated the basic effect multiple times. The simulation method revealed a similar robustness, finding the same effect in 80% of samples sized *N* = 30. Effectively equivalent, again, to multiple replications in the same dataset. For the lexical interference effect, we see that the data are not so robust: The resampling method estimated 80% power with 40 participants—again, replicating the effect multiple times. But the simulation method disagreed, estimating 80% power with 170 participants. Thus, the simulation method suggests that we did not, in effect, replicate our own effect within the overall dataset (of *N* = 202). Which to believe? Given that the resampling method reflects our *actual* data, whereas the simulation method reflects an idealization of the data that is determined by how good a fit the statistical model is to those data, we go with the resampling method for estimating the power associated with *this particular dataset*. That is, the resampling method is a better reflection of the actual data we collected. It is quite another matter, however, to determine which method is the better one for estimating appropriate sample sizes in *future* experiments.

The resampling method relies on having data that have the same distribution as one anticipates obtaining in the study (for example, if running a replication of an existing study with existing data that one can resample). The simulation method is useful when there is no such existing dataset, and in which parameters that determined the original distribution might be different; a model that is more tolerant of differences from what will actually be obtained (i.e., the simulation method) may provide a more accurate estimate of required sample size.

In a follow-up study we collected an additional dataset with Russian heritage speakers in an English-dominant environment, a relatively small population, using the same experiment (i.e., stimuli and methods) as described here. We replicated the lexical interference effect with 40 participants, the threshold offered by the resampling approach. Thus, for our purposes, the resampling method appears to have provided a useful estimate of the minimum sample size required to observe an effect, whereas the estimate provided by the simulation method appears to have been overly conservative. Of course, we cannot be certain that the effect we observed in the Russian heritage speakers will replicate. But given that we anticipated data with a similar distributional profile to the current dataset (because the same stimuli and methods were employed, with fluent speakers of the same language, albeit heritage speakers), we can be reasonably confident, in the context of the current data and associated (resampling) power analyses, that it will. Had we relied on the simulation method for calculating power, we may never have even collected those 40 participants' worth of heritage speaker data. To be clear, we are not advocating more generally for or against either of these two power analyses as being the most appropriate. As with any statistical test, there is no “one size fits all.” In the way we collected data, the numbers of participants we were able to test from which we could resample our own data and the similarity between the heritage speakers and the current participants suggested that the less conservative estimate based on resampling would be sufficient. However, more specific, dedicated research into the generalizability, and under what conditions, of the different methods for power analysis is warranted. Appropriate statistical power is crucial to doing meaningful science, yet in our field ensuring one is not being overly conservative is of importance not least since all languages and peoples are not equally available, despite all being of equal value and importance.

Below, we consider a number of other parameters, beyond participant numbers, that can influence the accuracy and/or resolution of online eye tracking. Much like our analyses of power, each of these can impact the generalizability of the data:

### Sampling rate

As discussed above, the sampling rate in webcam-based eye tracking cannot exceed that of the maximum frame rate of a webcam, which is currently 60 Hz. However, in reality, as a few previous studies as well as ours have shown, the sampling rate is even smaller because it depends on the computational load of participants’ computers with 19.7 Hz being the average in our study. In comparison, Altmann and Kamide (1999) had a sampling rate of 250 Hz and Kukona et al. (2014) had a sampling rate of 60 Hz. As our study shows, temporal resolution of as low as 19.7 Hz is more than sufficient to detect not only large and highly replicable effects from tracker-based eye-tracking studies, but crucially more fine-grained, subtle language processing effects. A question that arises in relation to this observation is what is the minimally required sampling rate for VWP eye-tracking studies. The answer naturally depends on the design of one's study. If one is simply interested in comparing the number of looks/fixations to the target at a particular time point/window of interest as a function of a condition, the sampling rate as low as 20 Hz is sufficient, as our study shows. If, on the other hand, one is interested in the fine detail of the time course of the unfolding of the effect, then a higher sampling rate is advantageous because one wants to capture the precise time the fixation started and minimize the uncertainty about the behavior between the samples. Higher sample rates allow the researcher to distinguish fixations from saccades and to make more fine-grained estimates of when the planning of the eye movements is replaced by the execution of those movements. Similarly, the spatial configuration of the regions of interest can determine whether low sample rates will be sufficient—distinguishing between looks towards one quadrant or another requires a lower sampling rate than does distinguishing successive fixations to smaller regions of interest during visual search, for example.

### Sound onset lag

In the “Data preprocessing” section, we showed how much variability there is in audio onset times. Such variability was not explored in the three studies discussed in the Introduction; however, some other as-yet unpublished work (Langlois et al., 2023) also reports similar delays, accompanied by delayed experimental effects. Thus, it is likely that delays in fixations reported in that earlier work were caused in part by delays in audio onset times. Regardless of the software that one is using to run their experiments, access to a metric with actual onset times (as opposed to assuming that the onset times are as specified by the researcher) is essential if the intention is to synchronize the eye movement/gaze record with specific points in the audio playback. Our data do not allow us to conduct a systematic investigation addressing the source of the auditory lag issues; however, we refer the reader to some previous work (Bridges et al., 2020) which looked at the variability in auditory and visual stimuli timing as a function of experimental package, browser, and operating system.

### Calibration

In our study, we chose to calibrate at the beginning of the study as well as after every 16 trials. Overall, 26 out of 253 participants (10.3%) who started the study did not pass the calibration. This relatively high number can be reduced by providing participants with detailed instructions about the lighting conditions required for the study. The instructions should convey the idea that good lighting is essential for participation in the study, and participants should plan this before starting the study. As for the frequency of recalibrations, there should be a balance between participants’ comfort (too many calibrations can exhaust them) and data quality. Given that the number of excluded trials due to low-quality predictions (according to the data quality metrics, “convergence” and “face_conf”) was very low (less than 0.1%), we consider our choice of recalibration every 16 trials to be good practice (although see Vos et al., 2022 for more recommendations on the piloting procedure for determining the optimal recalibration frequency).

### Timing

As noted above, timing delays in the presentation sequence are a potentially significant limitation of webcam-based eye tracking. Indeed, both Slim and Hartsuiker (2022) and Degen et al. (2021) reported significant delays in the emergence of the investigated effects relative to in-lab experimentation. It could be that some sacrifices in the time domain might be an inherent trade-off when adopting webcam-based eye tracking. We would suggest, however, that such trade-offs will not pose a limitation on webcam-based eye tracking: For example, although testing was a mere handful of months after the other studies referenced immediately above, Vos et al. (2022), using an updated version of WebGazer.js, found significantly reduced delays. Experimental design also factors into whether such tradeoffs need to be made: In the present study, for example, our main concern was to determine if webcam-based eye tracking could replicate a subtle effect which focused on looks at a single time point during each trial. Thus, while we have no grounds to speculate much about the temporal unfolding of the effect (but see Figs. 5B and 7B), the analysis revealed a qualitatively similar effect and, crucially, at the same time point as the original effects found in the original studies that we replicated. Although relative delays in the timing domain do exist, despite these, webcam-based eye tracking even in its present technological state is able to address and provide sound answers to a majority of questions that are asked within psycholinguistic VWP studies.

## Conclusions

In this study, we replicated two psycholinguistic effects: a robust verb semantic constraint effect first reported in Altmann and Kamide (1999) and a smaller effect of lexical interference first identified by Kukona et al. (2014). While some previous studies (Degen et al., 2021; Slim & Hartsuiker, 2022; Vos et al., 2022) have already reported webcam-based eye-tracking replications of effects tested in the lab, this is the first study to do so using the version of WebGazer.js implemented in Gorilla (and utilizing a fully GUI-based approach) and in a language other than English (Russian). This emerging bulk of evidence suggests that webcam-based eye tracking is not just a viable alternative to lab-based eye tracking, but in fact has advantages over lab-based eye tracking. Since it does not require a researcher to set up and oversee the experiment in person, it affords the recruitment of more diverse populations representing un(der)represented groups of people and/or specific languages, and for running experiments around the subjects' own dynamic scheduling. Moreover, given the significant reductions in (capital equipment) costs and required infrastructure, webcam-based eye tracking is more accessible to researchers who do not have their own dedicated lab space or the means to afford the high costs of equipment and personnel related to tracker-based eye tracking. Lowering the bar to accessibility in such ways can simultaneously address an additional problem in psycholinguistics research related to power: many—not all—psycholinguistic studies are underpowered; a problem that can be obviated by the increased accessibility and borderless reach of internet-mediated experimentation. Provided that measures are in place to ensure the quality of the data collected, the future of webcam-based eye tracking is bright.

## Appendix A

This appendix contains materials used in the eye-tracking experiment

Stimuli for testing the Anticipation effect

1. Девочка почистит/возьмет банан.
  The girl will peel/take the banana.
2. Женщина выжмет/купит лимон.
  The woman will squeeze/buy the lemon.
3. Парень погасит/нарисует сигарету.
  The young man will put out/draw the cigarette.
4. Доктор пропишет/подготовит таблетки.
  The doctor will prescribe/prepare the pills.
5. Мужчина расчешет/рассмотрит бороду.
  The man will comb/inspect the beard.
6. Женщина растопит/отдаст шоколадку.
  The woman will melt/give away the chocolate bar.
7. Путешественник разведет/сфотографирует костер.
  The hiker will start/photograph the fire.
8. Женщина полъет/подвинет растение.
  The woman will water/move the plant.
9. Девушка застелит/обойдет кровать.
  The young woman will make/walk around the bed.
10. Парень расстегнет/продаст пальто.
  The guy will unzip/sell the coat.
11. Бабушка распутает/спрячет клубок.
  The grandmother will untangle the ball of yarn.
12. Мужчина пролистает/одолжит газету.
  The man will flip through/borrow the newspaper.
13. Девочка подпишет/выберет открытку.
  The girl will sign/choose the postcard.
14. Мужчина срубит/увидит дерево.
  The man will cut/see the tree.
15. Мужчина построит/потрогает стену.
  The man will build/touch the wall.
16. Женщина пришьет/выбросит пуговицу.
  The woman will sew on/throw away the button.

Stimuli for testing the Lexical Interference effect

1. Мальчик словит этот белый/розовый мяч.
  The boy will catch this white/pink ball.
2. Студент соберет этот красный/желтый рюкзак.
  The student will pack this red/yellow backpack.
3. Женщина выпьет этот желтый/белый напиток.
  The woman will drink this yellow/white beverage.
4. Женщина порежет этот зеленый/оранжевый перец
  The woman will chop this green/orange bell pepper.
5. Бабушка свяжет этот красный/серый свитер.
  The grandmother will knit this red/grey sweater.
6. Девочка развернет этот зеленый/красный подарок.
  The girl will unwrap this green/red gift.
7. Мужчина зашнурует этот коричневый/серый ботинок.
  The man will lace this brown/grey boot.
8. Женщина сорвет этот розовый/желтый цветок.
  The woman will pluck this pink/yellow flower.
9. Женщина выгуляет эту белую/коричневую собаку.
  The woman will walk this white/brown dog.
10. Мужчина заведет эту красную/синюю лодку.
  The man will start this red/blue boat.
11. Женщина прочитает эту синюю/коричневую книгу.
  The woman will read this blue/brown book.
12. Мужчина пожарит эту серую/красную рыбу.
  The man will fry this grey/red fish.
13. Дедушка выкурит эту черную/коричневую трубку.
  The grandfather will smoke this black/brown pipe.
14. Мальчик наклеит эту розовую/оранжевую марку.
  The boy will stick this pink/orange stamp.
15. Женщина застегнет эту желтую/зеленую куртку.
  The woman will zip this yellow/green jacket.
16. Женщина наполнит эту голубую/красную миску.
  The woman will fill this blue/red bowl.
17. Мальчик съест этот коричневый/белый торт.
  The boy will eat this brown/white cake.
18. Мужчина откроет этот серый/желтый замок.
  The man will open this grey/yellow lock.
19. Мальчик надует этот оранжевый/зеленый шарик.
  The boy will inflate this orange/green balloon.

20. Женщина постирает этот розовый/коричневый шарф.
  The woman will wash this pink/brown scarf.
21. Мужчина завяжет этот синий/красный галстук.
  The man will tie this blue/red tie.
22. Девочка заточит этот зеленый/желтый карандаш.
  The girl will sharpen this green/yellow pencil
23. Мужчина запечатает этот синий/зеленый конверт.
  The man will seal this blue/green envelope.
24. Женщина пропылесосит этот желтый/голубой ковер.
  The woman will vacuum this yellow/blue carpet.
25. Женщина наденет эту коричневую/белую юбку.
  The woman will put on this brown/white skirt.
26. Мужчина погладит эту белую/черную рубашку.
  The man will iron this white/black shirt.
27. Девушка зажжет эту желтую/розовую свечу.
  The girl will light up this yellow/pink candle.
28. Женщина подоит эту коричневую/белую корову.
  The woman will milk this brown/white cow.
29. Мальчик разрушит эту оранжевую/зеленую башню.
  The boy will ruin this orange/green tower.
30. Девочка обнимет эту белую/розовую куклу.
  The girl will hug this white/pink doll.
31. Женщина нажмет эту зеленую/синюю кнопку.
  The woman will push this green/blue button.

32. Мужчина настроит эту красную/синюю гитару.

        The man will tune this red/blue guitar.

## Footnotes

1 There are two main paradigms for eye tracking in psycholinguistic research: the reading paradigm and the visual world paradigm (VWP). Reading research requires highly sensitive devices, which should be able to track people’s gaze with letter-by-letter accuracy (Kaiser, 2013). As a result, it is not surprising that reading studies have not (yet), to the best of our knowledge, been attempted with webcam-based eye tracking. Thus, by eye tracking in the present paper we are referring to the VWP.

2 Accuracy (where the eye is fixating) and resolution (how far the eyes need to move for the change in fixation to be recognized) are distinct measures. Resolution (in conjunction with sampling rate) is critical to recognizing saccades and establishing the time course of saccades and fixations. Accuracy in the VWP (unlike in studies of eye movement control during reading) is somewhat less critical.

3 WebGazer.js is one of several existing libraries for webcam-based eye tracking, and Gorilla is just one platform which utilizes it for online eye tracking. For other resources and helpful discussions on various aspects of them, see Vos et al. (2022).

4 These computations were necessary for applications requiring gaze-contingent feedback but not for the kinds of experiment reported by Yang and Kraibich or the kinds of VWP studies discussed here.

5 The original study was a cross-linguistic study comparing the timing of activation of representational content of perfectivity in Russian, English, and Spanish, which tends to be expressed differently among these three languages, and as a consequence structure the flow of events in ways that are not easily captured by offline methods.

6 Gorilla’s Eye Tracking 2 “zone has been using a modified version of WebGazer since November 11, 2021, which resolves the [timing] issue” (email from Gorilla support team). For a more technical discussion of these changes, see Gorilla release notes from November 11, 2021, on https://support.gorilla.sc/support/release-notes#releasenotes.

7 There were no determiners in the stimuli used to replicate the anticipation effect, however materials used to replicate the lexical interference effect did contain modifying demonstratives. See Appendix A for the full list of stimuli.

8 Is it not obvious why some participants failed calibration. One relevant observation is that of the 220 participants who made it through calibration, 127 participants had a Chrome browser, 51 Yandex, 11 Firefox, 21 Opera, and 10 Edge. None of the participants had a Safari browser. A closer look at rejected participants showed that calibration failed in Chrome, Edge, Firefox, Opera, Yandex, and Safari, and also in both Mac and Windows. A good rule of thumb in any case is to test your experiment in different browsers and take the results of your tests into account when setting filters for recruitment (Gorilla allows researchers to limit device browsers).

9 Note that this is twice the number of stimuli used in the original study by Kukona et al. (2014). This is because there was an additional manipulation of grammatical gender described further in the manuscript.

10 Since it usually takes around 200 ms to initiate a saccade (Matin et al., 1993; with some other work suggesting that it is possible to launch an eye movement as a function of a linguistic stimulus as early as 100 ms, see Altmann, 2011), information recorded at 99.9 ms (the latest first sample after the noun onset in our dataset) still reflects activation as a function of previous linguistic input.

11 Since there is some disagreement in the field regarding the empirical logit transformation (Donnelly, & Verkuilen, 2017), we also ran a generalized linear mixed-effects model on binomial data with preserved trial-level information (with 1s indicating that there was a look to the target and 0s indicating that there was no look). The effect of verb type was still significant. We did not try this approach for modeling the lexical interference effect because in that subset of data, the area of interest (i.e., the type of distractor) was in fact the condition (same or different). Given that both types of distractors were present in the same picture, the looks to either of them were not independent from the looks to the other. To overcome this violation of the assumption of independence of data points, one would normally compute some sort of an advantage score (for example, the log ratio, Ito & Knoeferle, 2022). In our data, we cannot do that because it would absorb our condition. This motivates our choice of the dependent variable.

12 In the present study we estimated power only for varying sample sizes. However, it is also important to know how power would change as a function of the number of items. Here, we could not manipulate it given that we aggregated across trials to compute our dependent variable, so it remains an important question for further exploration. The existing software (for example, the *mixedpower* package) does allow for varying both the number of subjects and items in the same simulation.
